# Fecal Microbiota Transplantation for Severe Infant Botulism, China

**DOI:** 10.3201/eid3008.231702

**Published:** 2024-08

**Authors:** Chaonan Fan, Rubo Li, Lijuan Wang, Kechun Li, Xinlei Jia, Hengmiao Gao, Bike Zhang, Xuefang Xu, Suyun Qian

**Affiliations:** Beijing Children's Hospital, Capital Medical University, National Center for Children's Health, Beijing, China (C. Fan, R. Li, L. Wang, K. Li, X. Jia, H. Gao, S. Qian);; National Key Laboratory of Intelligent Tracking and Forecasting for Infectious Diseases, National Institute for Communicable Diseases Control and Prevention, Center for Disease Control and Prevention, Infectious Disease Prevention and Control Institute, Beijing (B. Zhang, X. Xu)

**Keywords:** Infant botulism, Clostridium botulinum, fecal microbiota transplantation, source-tracking, gut microbiome, targeted metabolomics, bacteria, China

## Abstract

Infant botulism in a 4-month-old boy in China who continued to excrete toxins for over a month despite antitoxin therapy was further treated with fecal microbiota transplantation. After treatment, we noted increased gut microbial diversity and altered fecal metabolites, which may help reduce intestinal pH and enhance anti-inflammatory capabilities.

Infant botulism is caused by ingesting *Clostridium botulinum* spores and characterized by symmetric descending paralysis, which can progress to respiratory failure in severe cases (*1*). Specific therapies include intravenous administration of botulism immune globulin (BIG-IV or BabyBIG, https://infantbotulism.org/general/babybig.php) or botulinum antitoxin. However, BIG-IV and BabyBIG are unavailable in some countries, including China (*2*). Even after clinical signs have been alleviated through antitoxin therapy, some children may continue to excrete *C. botulinum* and its neurotoxin (BoNT) in their feces over a prolonged period, heightening the potential for relapse and transmission to others (although relatively rare) (*3*,*4*). Hence, effective treatments that promote clearance of intestinal *C. botulinum* spores are needed. During March–May 2021, we treated severe infant botulism in a 4-month-old boy in China who had continued excreting toxins for >1 month after clinical signs disappeared after antitoxin therapy. The study was approved by the Ethics Committee of Beijing Children’s Hospital (2023-E-149-R).

Five days before admission to Beijing Children’s Hospital (Beijing, China), the previously healthy infant exhibited intermittent fever, lethargy, poor appetite, and constipation, followed by respiratory distress. After intubation and mechanical ventilation in the emergency department, the patient was admitted to the pediatric intensive care unit. During examination, his pupils were dilated (≈4 mm) and had sluggish light reflexes but no signs of meningeal irritation. In addition, his muscle strength and tone were low. A series of tests excluded central nervous system infections, metabolic disorders, and other potential causes. By day 3 of hospitalization, the diagnosis of infant botulism was confirmed by detection of BoNT nucleic acid (serotype B) in fecal samples. Subsequently, we were able to obtain botulism antitoxin (monovalent type B) and administer it by intravenous injection of 2 mL (5,000 IU) 2×/day. Substantial improvement in clinical signs was observed by day 7 of hospitalization, and complete resolution was achieved by day 15.

Nevertheless, through day 33, multiple fecal samples tested for botulinum nucleic acid by real-time PCR and for BoNT by mouse bioassays (*5*) remained positive (Table). Consequently, during days 34–37 of hospitalization, the infant received a fecal microbiota transplantation (FMT; donor identification D024) at a dose of 20 mL via rectal enema daily for 4 consecutive days. During that time, he experienced low-grade fever and mild coughing, but his body temperature returned to reference range after FMT. After the third FMT (on day 36 after admission), test results for BoNT nucleic acid and mouse bioassays in feces were all negative (Table). 

**Table:** Analysis of *Clostridium botulinum* and botulinum neurotoxin in 4-month-old boy with infant botulism, Beijing, China*

Fecal sample no.	Days after admission	Sample classification	Real-time PCR result	Isolated strain	Mouse bioassay result
A1	3	Before FMT	BoNTB	B	BoNTB
A2	23	Before FMT	BoNTB	B	BoNTB
A3	27	Before FMT	BoNTB	B	BoNTB
A4	32	Before FMT	BoNTB	B	BoNTB
A5	33	Before FMT	BoNTB	B	BoNTB
A6	34	FMT1	Negative	B	Negative
A7	36	FMT3	Negative	Negative	Negative
A8	37	FMT4	Negative	Negative	Negative
A9	38	After FMT	Negative	Negative	Negative
A10	39	After FMT	Negative	Negative	Negative
A11	182	Follow-up	Negative	Negative	Negative

*BoNTB, *Clostridium botulinum* neurotoxin B; FMT, fecal microbiota transplantation.

We conducted epidemiologic investigations for the exclusively breastfed patient and isolated *C. botulinum* from his home environment; serologic and genetic types matched those in his feces. After discharge, he was followed up for 6 months and exhibited good growth and development without any relapse. 

We analyzed gut microbiota composition and fecal metabolomics of 10 fecal samples (5 time points before and after FMT) (Figure; Appendix Figure 1). Use of 16S rRNA sequencing revealed increased α diversity of the gut microbiota after FMT, indicated by the Abundance-based Coverage Estimator and Shannon indices (*6*). At the genus level, relative abundance of *Enterococcus* and *Lactobacillus* was decreased and that of *Collinsella* and *Holdemanella* was increased. Although 16S rRNA sequencing cannot directly detect *C. botulinum*, the taxonomic group to which *C. botulinum* belongs (*Clostridium_sensus_stricto_18*) was also reduced. Ultra-high performance liquid chromatography–tandem mass spectrometry further revealed statistically significant alterations in 53 fecal metabolites after FMT; primarily implicated metabolic pathways were α-linolenic acid and linoleic acid metabolism, bile acid biosynthesis, bile acid metabolism, branched-chain amino acid metabolism, and butyrate metabolism. Correlation analyses indicated that changes in those metabolites were closely associated with alterations in the gut microbiota (Appendix Figure 2).

**Figure:**
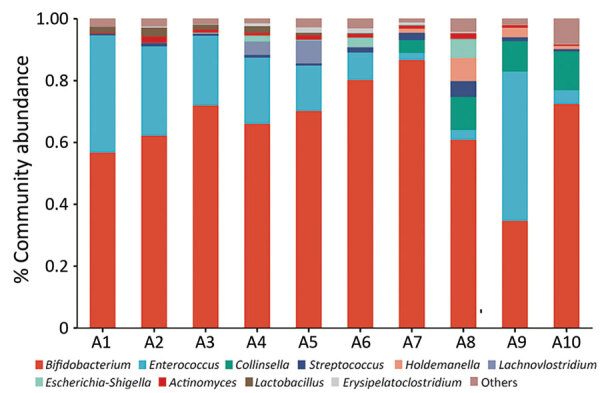
Relative gut microbiota abundance at the genus level before and after fecal microbiota transplantation (FMT) in 4-month-old boy with infant botulism, Beijing, China. The collected fecal samples (A1–A10) included 5 samples before FMT (A1–A5), 3 samples during FMT (A6–A8), and 2 samples after FMT (A9–A10), as shown in the Table.

Infants with infant botulism may continue to excrete *C. botulinum* and BoNT for weeks or even months (*7*). The simpler gut microbiota with fewer taxa and the deficiency of bile acids in infants might contribute to the overgrowth of *C. botulinum* spores and their persistent excretion in the intestine (*8*). Our study illustrated increased gut microbiota diversity after FMT, concurrently accompanied by increased bile acid, potentially shifting the function of the microbiota–bile acid axis (*9*). Moreover, the increased production of metabolites, such as organic acids and docosapentaenoic acid, may help reduce intestinal pH and enhance anti-inflammatory capabilities (*10*). However, whether the restoration of gut microbiota diversity and changes in fecal metabolites are directly associated with the disappearance of *C. botulinum* spores is not clear. Because our study lacked a control group and solely represents an observational phenomenon, we cannot provide causal evidence for the effectiveness of FMT in treating infant botulism. Interventions such as fecal transplantation are recommended for children with infant botulism who continue to produce toxins for a long time.

Appendix Additional information on study of fecal microbiota transplantation for severe infant botulism.
