# Supplementary material for: Fecal Microbiota Transplantation for Severe Infant Botulism, China
Source: Emerg Infect Dis. 2024 Aug;30(8):1732–4. doi: 10.3201/eid3008.231702 (PMC11286044; doi:10.3201/eid3008.231702)
Supplement: Appendix — Additional information on study of fecal microbiota transplantation for severe infant botulism. [file 23-1702-Techapp-s1.pdf]

Article DOI: https://doi.org/10.3201/eid3008.231702

EID cannot ensure accessibility for supplementary materials supplied by authors. Readers who have difficulty accessing supplementary content should contact the authors for assistance.

Fecal Microbiota Transplantation for Severe Infant Botulism, China

Appendix

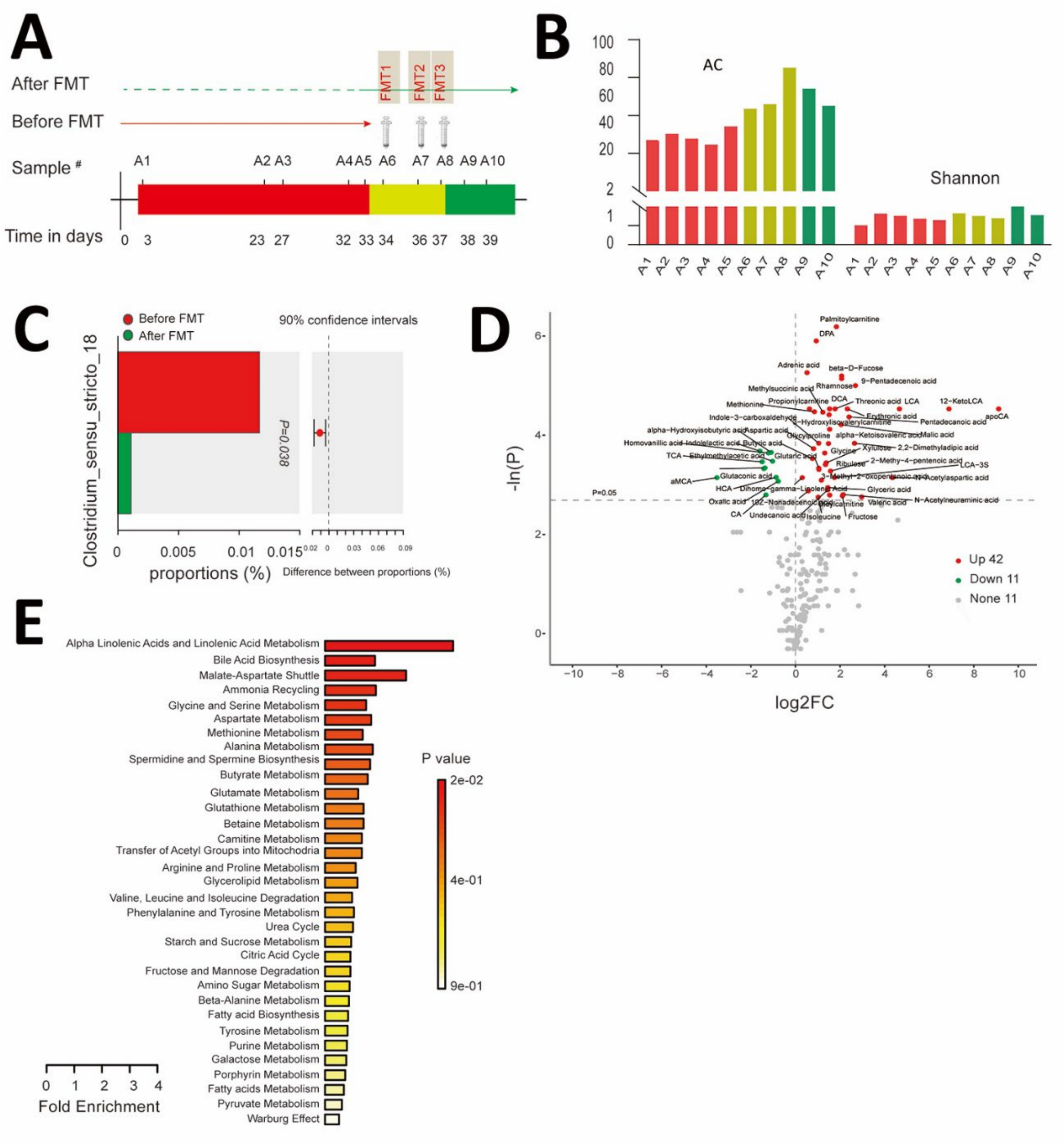

Appendix Figure 1. Structural modulation of gut microbiota and changes in its metabolites in a patient with infant botulism after FMT. A) Flowchart showing collection of samples at 10 time points, including 5 samples before

FMT (red), 3 samples during FMT (yellow), and 2 samples after FMT (green). B) ACE and Shannon indices, measures of α diversity, were found to be higher after FMT. C) Relative abundance rates of Clostridium_sensu_stricto_18 significantly decreased after FMT. D) Volcano plot showing differentially accumulated metabolites before and after FMT. Of the identified metabolites, 53 showed statistically significant changes: 42 increased and 11 decreased after FMT. E) Pathway enrichment and statistical significance of the 42 metabolites unique to the after FMT periods. ACE, Abundance-based Coverage Estimator; FMT, fecal microbiota transplantation.

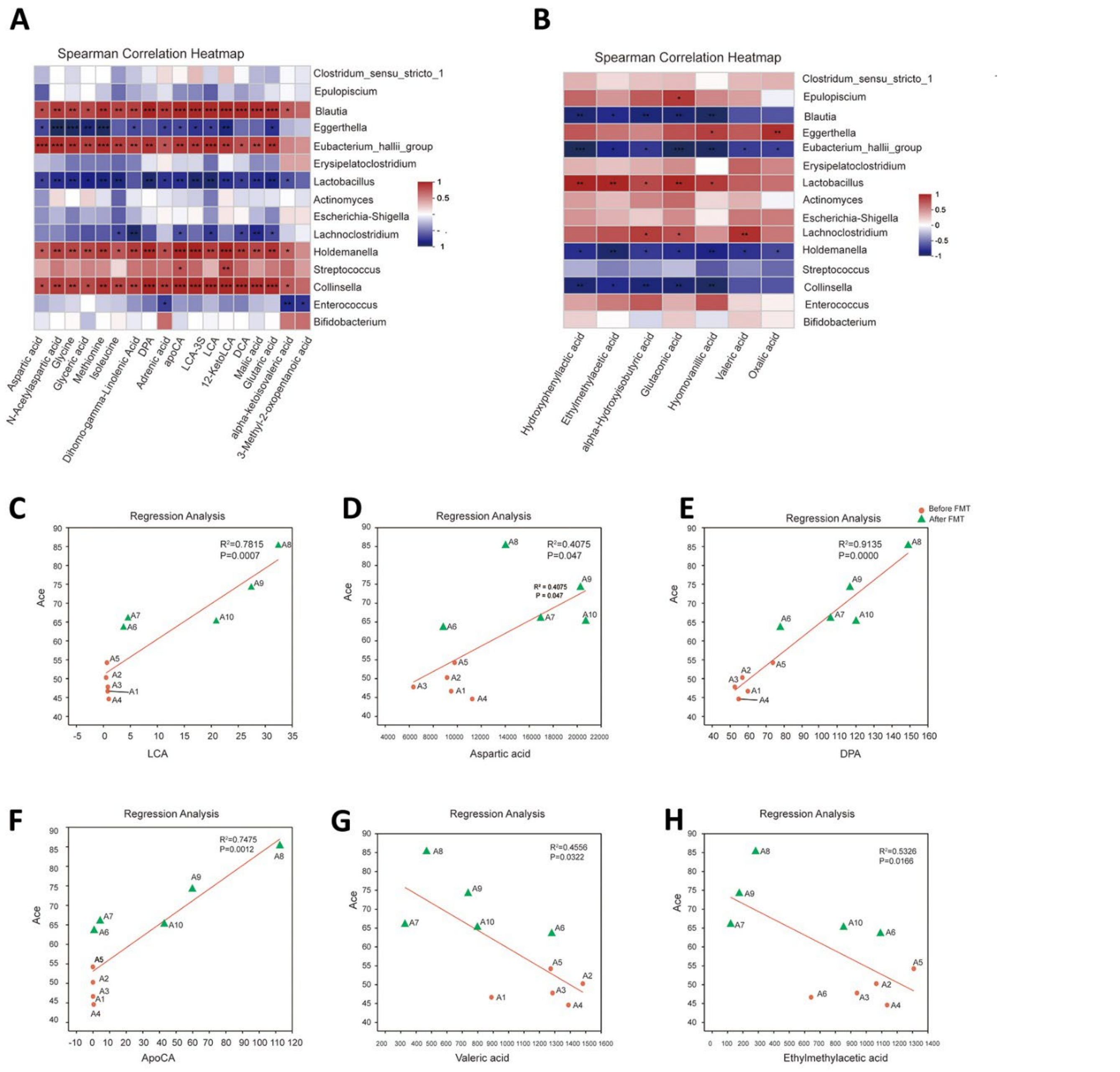

Appendix Figure 2. Cross-correlation analysis between gut microbiota and fecal metabolites**.** (A) Up-regulated metabolites after FMT. (B) down-regulated metabolites after FMT. (C–H) Scatter plot of the correlation between

six metabolites (LCA, aspartic acid, DPA, ApoCA, valine, Ethylmethylacetic acid) and the ACE index, α-diversity of gut microbiome. BF, before FMT; AF, after FMT; FMT, fecal microbiota transplantation.
